# Regulation of hepatitis B virus replication by epigenetic mechanisms and microRNAs

**DOI:** 10.3389/fgene.2013.00202

**Published:** 2013-10-14

**Authors:** Xiaoyong Zhang, Jinlin Hou, Mengji Lu

**Affiliations:** ^1^Hepatology Unit and Department of Infectious Diseases, Nanfang Hospital, Southern Medical UniversityGuangzhou, China; ^2^Institute of Virology, University Hospital of Essen, University of Duisburg EssenEssen, Germany

**Keywords:** hepatitis B virus, microRNA, epigenetic regulation, histone deacetylases, DNA methyltransferase

## Abstract

The hepatitis B virus (HBV) genome forms a covalently closed circular DNA (cccDNA) minichromosome that persists in the nucleus of virus-infected hepatocytes. HBV cccDNA serves as the template for viral mRNA synthesis and is subject to epigenetic regulation by several mechanisms, including DNA methylation and histone acetylation. Recently, microRNAs (miRNAs), a class of small non-coding RNAs, were also directly connected to the epigenetic machinery through a regulatory loop. Epigenetic modifications have been shown to affect miRNA expression, and a sub-group of miRNAs (defined as epi-miRNAs) can directly target effectors of the epigenetic machinery. In this review, we will summarize recent findings on the epigenetic mechanisms controlling HBV cccDNA function, primarily focusing on the epi-miRNA functions operating in HBV replication. Investigation of the epigenetic regulation of HBV replication may help to discover novel potential therapeutic targets for drug development with the goal to eradicate the HBV cccDNA pool in hepatocytes.

## INTRODUCTION

Hepatitis B virus (HBV) infection is a global health problem that causes a wide spectrum of liver diseases, including acute or chronic HBV infection. Acute HBV infections either resolve or progress to chronicity. Chronic hepatitis B (CHB) is associated with chronic hepatitis, cirrhosis, and hepatocellular carcinoma (HCC; McMahon, 2009). It is estimated that more than 350 million patients worldwide are chronically infected with HBV, with the majority of these patients living in the Asia-Pacific region. More than one million deaths occur each year as a direct consequence of CHB (Dienstag, 2008). Medical intervention using antiviral nucleoside/nucleotide analogs and interferon (IFN) was established to treat chronically infected patients (Pardo et al., 2007). However, currently available therapies do not lead to the termination of HBV infection in the majority of patients (Mailliard and Gollan, 2006). There is a consensus that the improved understanding of the HBV–host interaction is a prerequisite for new antiviral therapeutic strategies. Recently, many aspects pertaining to the epigenetic mechanisms responsible for viral persistence and clearance during HBV replication have been addressed, including methylation of viral DNA, acetylation of histone complexes, and microRNA (miRNA) regulation. These topics are described in this review.

## HBV cccDNA STRUCTURE AND ITS ROLE IN HBV INFECTION

Hepatitis B virus is the prototype member of the family Hepadnaviridae and has a partially double-stranded DNA genome of approximately 3.2 kb in length. The viral genome harbors seven open reading frames, coding for the viral polymerase, HBV core, and e antigens (HBcAg and HBeAg); the regulatory HBx protein; and the preS/S gene encoding the three surface antigens (LHBsAg, MHBsAg, and SHBsAg). The genome also contains a number of regulatory elements (Seeger and Mason, 2000). The entry of HBV virions is likely initiated through a non-specific interaction with negatively charged glycans at the surface of hepatocytes (Schulze et al., 2007; Bremer et al., 2009) followed by specific binding to the sodium-taurocholate cotransporting polypeptide (NTCP) receptor by a specific sequence (2-48aa) located in the preS1 domain of the LHBsAg protein (Yan et al., 2012). After uncoating, the HBV capsid is transported by the cellular machinery to the nuclear pore. The open circular form of HBV genomic DNA is then converted to a covalently closed circular DNA (cccDNA) molecule in the nucleus. This process requires that the covalently attached viral polymerase is removed from the negative DNA strand by a proteinase and that the positive strand DNA is completed by the cellular replicative machinery so that it matches the negative strand to covalently join the two ends to form a circular, supercoiled molecule (Gao and Hu, 2007).

In the nucleus, HBV cccDNA is incorporated into the host chromatin and exists as an individual minichromosome with a “beads-on-a-string” structure, which is revealed by electron microscopy (Bock et al., 1994; Newbold et al., 1995). This minichromosome has been shown to consist of both histone and non-histone proteins. By immunoblotting with HBcAg, the histone proteins H3 and H2B were the most prominent species, while lower levels of H4, H2A, and H1 were also detectable (Bock et al., 2001). Using the cccDNA-ChIP assay, the group of Massimo Levrero has confirmed the recruitment of the H3 and H4 histones along with the HBcAg and HBx proteins to the cccDNA minichromosome. Using the same approach, several cellular transcription factors (CREB, ATF, YY1, STAT1, and STAT2) and chromatin modifying enzymes (PCAF, p300/CBP, HDAC1, SIRT1, and EZH2) have been shown to bind to the cccDNA in human hepatoma cells containing replicating HBV (Pollicino et al., 2006; Belloni et al., 2009, 2012). The histone acetyltransferases (HATs) p300/CBP and PCAF and the histone deacetylases (HDACs) HDAC1 and SIRTl were shown to be recruited with different kinetics onto HBV cccDNA, implying that HBV cccDNA-bound histones may be subjected to regulatory post-translational modifications (Levrero et al., 2009).

Because cccDNA is the transcriptional template of the virus (Quasdorff and Protzer, 2010), it is required for the maintenance of HBV infection. Unlike HBV transcripts and replicative intermediates, cccDNA is very stable in quiescent hepatocytes and is responsible for the persistence of infection during the natural course of chronic HBV infection and during prolonged antiviral therapy (Werle-Lapostolle et al., 2004). The cccDNA may persist for many years in the liver of patients, even after successful antiviral treatment and reinforcement of immunologic control (Zoulim, 2005). Currently, little is known about the mechanism of HBV cccDNA maintenance in the nuclei of hepatocytes. However, it has been shown that the cccDNA can be eliminated when infected hepatocytes are removed by immune cell-mediated killing or other non-cytopathic mechanisms (Murray et al., 2005) and replaced by cell turnover (Lutgehetmann et al., 2010).

## REGULATION OF HBV cccDNA TRANSCRIPTION BY EPIGENETIC MODIFICATION

### HISTONE ACETYLATION AND METHYLATION

Recently, it was proposed that the functionality of HBV cccDNA might be controlled by epigenetic mechanisms, regulating its transcriptional activity and HBV replication. Histones and non-histone proteins either bind directly to the cccDNA or are indirectly recruited to viral minichromosomes through protein–protein interactions. Thereby, the acetylation and deacetylation of cccDNA-bound histones may regulate HBV transcription. Exploring a ChIP assay using anti-acetylated-H3 or -H4 antibodies, Pollicino et al. (2006) found that HBV replication is indeed regulated by the acetylation status of H3/H4 histones bound to the viral cccDNA, both in cell-based replication systems and in the liver of chronically HBV infected patients. The co-recruitment of PCAF and p300/CBP parallels viral replication *in vitro*, whereas HDAC1 recruitment onto the HBV cccDNA correlates with low HBV replication *in vitro* and with low viremia *in vivo*. The importance of epigenetic modifications of cccDNA-bound histones in the regulation of HBV replication is further confirmed by experiments exploring the class I and class III HDAC inhibitors trichostatin A (TSA), valproate, and nicotinamide (NAM). These HDAC inhibitors induce an evident increase of both cccDNA-bound acetylated H4 and HBV replication. Another study demonstrated a similar role for the acetylation of cccDNA-bound histones, as well as a role for methylation and phosphorylation of these proteins (Gong et al., 2011).

A recent study demonstrated that in cultured hepatoma cells with HBV replication and in mouse models with repopulated human hepatocytes, administration of IFN-α resulted in the active recruitment of the transcriptional corepressors HDAC1, SIRT1, and polycomb repressor complexes 2 (EZH2 and YY1) to HBV cccDNA as well as the hypoacetylation/hypermethylation of cccDNA-bound histones. IFN-α treatment also reduced the binding of the transcription factors STAT1 and STAT2 to the IFN-sensitive response element on active cccDNA (Belloni et al., 2012). These observations suggested that IFN-α could epigenetically regulate HBV replication, and the hypoacetylation/hypermethylation of histones was associated with decreased replication of HBV. Furthermore, it was shown that small molecules that inhibit p300 and PCAF or activation of SIRT1/2 and EZH2 could induce an “active epigenetic suppression” of the HBV cccDNA minichromosome to suppress HBV replication (Palumbo et al., 2013).

### HBV DNA METHYLATION

In addition to post-translational modification of histones, methylation of the CpG islands on HBV genomic DNA also contributes to the regulation of HBV gene expression (Mogul et al., 2011; Rivenbark et al., 2012). It has been shown that early integrated HBV DNA is methylated in HCC cells (Miller and Robinson, 1983; Chen et al., 1988). The non-integrated HBV DNA (Vivekanandan et al., 2008b) and cccDNA (Guo et al., 2009) could also be methylated in liver tissues from patients. Currently, at least six CpG islands have been identified in the HBV genome, including three conventional regions overlapping the start site of the HBV S gene (island 1), the region encompassing enhancer I and the X gene promoter (island 2), and the Sp1 promoter and start codon of the P gene (island 3; Zhang et al., 2013b). Methylation of CpG islands 1 and 2 was found in HBV DNA extracted from liver biopsies from CHB patients, suggesting that increased methylation of HBV DNA may decrease the production of viral proteins (Vivekanandan et al., 2008b). The hypermethylation of island 2 was correlated with low levels or absence of HBsAg production (Vivekanandan et al., 2008a), as well as reduced HBeAg expression (Guo et al., 2009). It was shown that individuals with occult HBV infection, which is characterized by the persistence of HBV DNA in the liver of individuals who test negative for the HBsAg, had a higher degree of methylation in island 2 compared to non-occult CHB patients (Vivekanandan et al., 2008a). Another study with a cohort of cirrhosis patients did not find an association between the methylation status of HBV cccDNA and HBsAg expression in liver tissues, but confirmed that a higher methylation density was associated with lower viral load, lower RNA copies per cccDNA, and lower virion productivity (Kim et al., 2011).

Consistent with these findings, transfection of methylated HBV DNA in HepG2 cells resulted in reduced HBV mRNA levels, decreased intracellular HBsAg and core HBcAg expression, and decreased secretion of HBV viral proteins into cell supernatants. Furthermore, an *in vitro* equivalent of cccDNA showed decreased viral protein production in HepG2 cells after DNA methylation (Vivekanandan et al., 2009). After transfection of HBV DNA into HepG2 cells, an inverse relationship between methylated HBV DNA and viral mRNA levels was observed in dependence on the upregulation of host DNA methyltransferase (DNMT). Cotransfection with DNMT3a and HBV DNA was associated with decreased production of HBsAg and HBeAg, as well as host proteins implicated in carcinogenesis (Vivekanandan et al., 2010). These data from cell culture experiments suggest that HBV DNA methylation is associated with down regulation of viral protein production.

## INTERPLAY BETWEEN HBV, miRNAs, AND THE EPIGENETIC MACHINERY

### miRNAs PLAY A PIVOTAL ROLE IN THE EPIGENETIC REGULATION NETWORK

MicroRNAs are approximately 22 nucleotide-long non-coding RNAs that are emerging as key players in regulating gene expression in eukaryotes, influencing various biological processes such as development, infection, immunity, and carcinogenesis (Ambros, 2004). The biogenesis and mechanisms of action of these tiny but potent molecules have been described in detail (Bartel, 2004). Briefly, miRNAs are transcribed from the host genome and generated by Drosha- and Dicer-mediated enzymatic cleavage. Mature miRNAs are engaged in either translational arrest or degradation of targeted transcripts through imperfect base pairing with the 3′-untranslated region (UTR) or the coding region of the target transcripts. Currently, more than 2000 miRNAs have been identified in human organs (Griffiths-Jones et al., 2008). The expression profiles of these miRNAs in different cells or tissues may exhibit temporal or tissue-specific patterns (Skalsky and Cullen, 2010).

Many studies have shown that a set of miRNAs play a pivotal role in the epigenetic regulation network (Chuang and Jones, 2007; Iorio et al., 2010). Epigenetic modifications, such as promoter methylation or histone acetylation, have been demonstrated to affect miRNA expression and are potentially responsible for the aberrant miRNA regulation observed in cancer (Baer et al., 2013). Along with the epigenetic regulation of miRNA expression, many miRNAs themselves can regulate the expression of components of the epigenetic machinery, creating a highly controlled feedback mechanism. A number of the miRNAs related to epigenetic regulation were defined as so-called “epi-miRNAs.” For example, DNMT1 overexpression was responsible for the hypermethylation of the miR-148a and miR-152 promoters. As a direct target of miR-148a and miR-152, DNMT1 was inversely related to the expression levels of miR-148a and miR-152 (Chen et al., 2013). Similarly, miR-1 and miR-449a, which could be induced by 5-AzaC/TSA treatment (Datta et al., 2008) or by HDAC1-3 knock down (Buurman et al., 2012) in HCC cells, directly targeted HDAC4 (Chen et al., 2006) and HDAC1 (Noonan et al., 2009), respectively.

### HBV INFECTION AFFECTS miRNA EXPRESSION

Although the viral miRNAs encoded by HBV have not been verified, the products of HBV were shown to alter miRNA expression profiles. In chronic HBV infection or HBV-related HCC, the miRNA profiles in liver tissue or serum levels from numerous studies are controversial and complicated (Ura et al., 2009; Hou et al., 2011; Liu et al., 2011; Wang et al., 2012b). For instance, it was reported that subviral HBsAg circulating in the blood of HBV carriers could carry liver-specific miRNAs (miR-27a, miR-30b, miR-122, miR-126, and miR-145) as well as immune regulatory miRNAs (miR-106b and miR-223) that were involved in hepatocarcinogenesis and HBV persistence (Novellino et al., 2012). In another study, three miRNAs (miR-122, miR-22, and miR-99a) were upregulated at least 1.5-fold in the sera of HBV-infected patients (Hayes et al., 2012). The highly liver-enriched, abundantly expressed miR-122 was consistently upregulated in HBV infected patients, and miR-145 could be used as a candidate tumor suppressive miRNA in the early steps of HBV-related hepatocarcinogenesis (Gao et al., 2011).

Recently, molecular studies have revealed that the HBx protein, which is essential for virus replication *in vivo*, induced epigenetic changes, including aberrations in DNA methylation, histone modifications, and miRNA expression. HBx expression has been found to be associated with alterations in the host miRNA profile through different epigenetic mechanisms (Tian et al., 2013). MiRNAs upregulated by HBx include miRNA-29a and miR-143 (Zhang et al., 2009; Kong et al., 2011). HBx-downregulated miRNAs include miR-101, miR-122, miR-132, miR-148a, miR-152, let-7, and the miR-16 family (Huang et al., 2010; Wang et al., 2010; Wu et al., 2011; Song et al., 2013; Wei et al., 2013a, b; Xu et al., 2013). In addition, HBx was shown to activate HBV transcription through opposition to the protein phosphatase 1 and HDAC1 complex on the HBV cccDNA (Cougot et al., 2012), or down-regulate DNMT3A expression through miR-101 induction (Wei et al., 2013b). Loss of HBx reduced recruitment of p300, caused rapid hypoacetylation of the cccDNA-bound histones and increased early recruitment of SIRT1 and HDAC1, accompanied by lower HBV replication (Belloni et al., 2009).

### CELLULAR miRNAs INHIBIT HBV REPLICATION BY DIRECT BINDING

**FIGURE 1:**
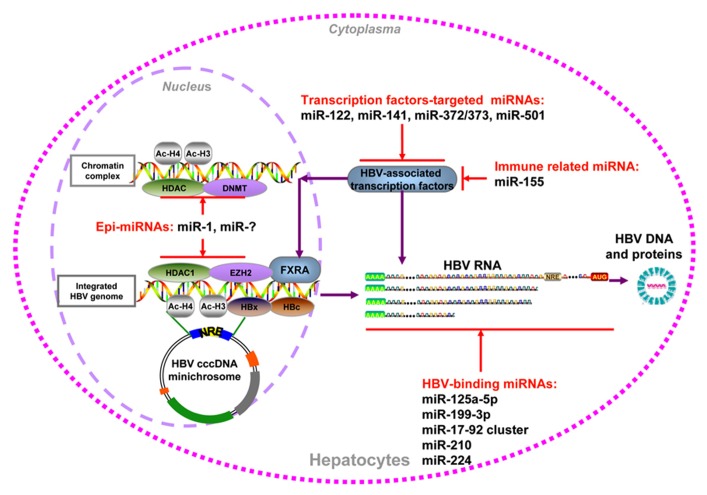
**Summary of cellular miRNAs effect on HBV replication.** HBV DNA could be integrated into host genome or form cccDNA minichromosome in the hepatocytes nucleus. It serves as the temple for viral transcription, viral DNA, and antigens production. The miRNAs which regulate HBV replication through epigenetic mechanism, transcription factors regulation, immune regulation, and direct targeting viral transcripts were indicated.

As HBV produces different transcripts during its life cycle, the transcripts are proposed to be targeted by cellular miRNAs. In a screen for cellular miRNAs affecting HBV replication, Zhang et al. (2010) employed a loss-of-function approach by transfecting antagomirs targeting 328 human miRNAs into HepG2 cells. Two miRNAs, miR-199a-3p and miR-210, were shown to suppress HBsAg expression. The direct effect of these two miRNAs on HBV RNA transcripts was validated by GFP reporter assay (Zhang et al., 2010). In addition, Russo’s group found that miR-125a-5p is able to interfere with HBsAg expression, thus reducing the amount of secreted HBsAg (Potenza et al., 2011). Recently, many cancer-related miRNAs, including miR-15a/miR-16-1 (Wang et al., 2013a), the miR-17-92 cluster (Jung et al., 2013), and miR-224 (Scisciani et al., 2011), were shown to target HBV mRNAs directly by luciferase reporter assay and inhibit HBV replication (summarized in **Figure 1**). Notably, the expression of these miRNAs was also linked to epigenetic regulation, as well as to promoter methylation (Dakhlallah et al., 2013) and histone acetylation (Zhang et al., 2013a; Wang et al., 2013b).

### CELLULAR miRNAs REGULATE HBV REPLICATION INDIRECTLY

In addition to direct targeting, some cellular miRNAs, including epi-miRNAs, were found to be capable of inhibiting or stimulating HBV replication by indirectly regulating cellular transcription factors. It was shown that the transcription of HBV cccDNA was tightly regulated by a number of liver-enriched transcription factors and nuclear receptors through the recognition of HBV promoter/enhancer elements (Quasdorff and Protzer, 2010). miR-122 may exert its effect on HBV indirectly via downregulation of its target cyclin G1, thus interrupting the interaction between cyclin G1 and p53 and abrogating p53-mediated inhibition of HBV replication (Wang et al., 2012a). miR-372 and -373 are upregulated in HBV-infected liver tissues and promote HBV gene expression through a pathway involving the transcription factor nuclear factor I/B (Guo et al., 2011). The higher expression of miR-501 in HCC tissues could enhance HBV replication partially by targeting HBXIP (Jin et al., 2013). In contrast, miR-141 significantly suppresses HBV expression and replication in HepG2 cells. Bioinformatic analysis and experimental assays indicate that peroxisome proliferator-activated receptor alpha is a relevant target of miR-141 during this process (Hu et al., 2013). For immune-related miRNAs, miR-155 enhances innate antiviral immunity by promoting the Janus kinase/signal transducer and activator of transcription (JAK/STAT) signaling pathway through the targeting of SOCS1, mildly inhibiting HBV infection in human hepatoma cells (Su et al., 2011).

By screening a set of cellular miRNAs, our group found that epigenetically regulated miR-1 over-expression resulted in a marked increase in HBV replication, accompanied with upregulated HBV transcription, antigen expression, and progeny secretion. HDAC4, the cellular target of miR-1, was able to suppress HBV replication. The expression of nuclear receptor farnesoid X receptor alpha (FXRA) was increased by miR-1, leading to the enhanced transcriptional activity of the HBV core promoter (Zhang et al., 2011). Furthermore, another epi-miRNA that targets HDAC1, miR-449a, had an even higher capacity for enhancing HBV replication but a lower level of induction of FXRA (Zhang et al., unpublished data). Additionally, both of these two defined epi-miRNAs could inhibit the G1/S cell cycle transition and promote cell differentiation by increasing the expression of hepatocyte-specific factors, which may be beneficial for HBV replication (Zhang et al., 2011). Collectively, host epi-miRNAs can modulate HBV replication by regulating cellular epigenetic factors or specific transcription factors that directly bind to the HBV cccDNA minichromosome (summarized in **Figure 1**).

## CONCLUSION AND PERSPECTIVE

In this review, we summarize the available information about the epigenetic mechanisms involved in the regulation of HBV cccDNA function. Notably, miRNAs could be considered part of a multilevel regulatory mechanism aimed to precisely modulate HBV replication and gene expression, likely in the response to the changing hepatic microenvironment. Considerably, many cellular miRNAs indirectly influence the HBV life cycle by regulating the expression of relevant cellular proteins and may play important roles in hepatitis B pathogenesis. Future studies need to be performed to elucidate the regulatory loop involving miRNAs and the cccDNA epigenetic machinery and certainly to investigate how to translate these findings into clinical applications.

## Conflict of Interest Statement

The authors declare that the research was conducted in the absence of any commercial or financial relationships that could be construed as a potential conflict of interest.

## Abbreviations

5-AzaC: 5-azacytidine
cccDNA: covalently closed circular DNA
CHB: chronic hepatitis B
DNMT: DNA methyltransferase
HBV: hepatitis B virus
HCC: hepatocellular carcinoma
HDAC: histone deacetylase
IFN: interferon
miRNA: microRNA
SIRT1: sirtuin 1
TSA: trichostatin A.

## References

[B1] Ambros V. (2004). The functions of animal microRNAs. *Nature* 431 350–355 10.1038/nature02871 15372042

[B2] Baer C. Claus R. Plass C. (2013). Genome-wide epigenetic regulation of miRNAs in cancer. *Cancer Res.* 73 473–477 10.1158/0008-5472.CAN-12-3731 23316035

[B3] Bartel D. P. (2004). MicroRNAs: genomics, biogenesis, mechanism, and function. *Cell* 116 281–297 10.1016/S0092-8674(04)00045-5 14744438

[B4] Belloni L. Allweiss L. Guerrieri F. Pediconi N. Volz T. Pollicino T. (2012). IFN-alpha inhibits HBV transcription and replication in cell culture and in humanized mice by targeting the epigenetic regulation of the nuclear cccDNA minichromosome. *J. Clin. Invest.* 122 529–537 10.1172/JCI58847 22251702 PMC3266786

[B5] Belloni L. Pollicino T. De Nicola F. Guerrieri F. Raffa G. Fanciulli M. (2009). Nuclear HBx binds the HBV minichromosome and modifies the epigenetic regulation of cccDNA function. *Proc. Natl. Acad. Sci. U.S.A.* 106 19975–19979 10.1073/pnas.0908365106 19906987 PMC2775998

[B6] Bock C. T. Schranz P. Schroder C. H. Zentgraf H. (1994). Hepatitis B virus genome is organized into nucleosomes in the nucleus of the infected cell. *Virus Genes* 8 215–229 10.1007/BF01703079 7975268

[B7] Bock C. T. Schwinn S. Locarnini S. Fyfe J. Manns M. P. Trautwein C. (2001). Structural organization of the hepatitis B virus minichromosome. *J. Mol. Biol.* 307 183–196 10.1006/jmbi.2000.4481 11243813

[B8] Bremer C. M. Bung C. Kott N. Hardt M. Glebe D. (2009). Hepatitis B virus infection is dependent on cholesterol in the viral envelope. *Cell. Microbiol.* 11 249–260 10.1111/j.1462-5822.2008.01250.x 19016777

[B9] Buurman R. Gurlevik E. Schaffer V. Eilers M. Sandbothe M. Kreipe H. (2012). Histone deacetylases activate hepatocyte growth factor signaling by repressing microRNA-449 in hepatocellular carcinoma cells. *Gastroenterology* 143 811–820 e15 10.1053/j.gastro.2012.05.033 22641068

[B10] Chen J. F. Mandel E. M. Thomson J. M. Wu Q. Callis T. E. Hammond S. M. (2006). The role of microRNA-1 and microRNA-133 in skeletal muscle proliferation and differentiation. *Nat. Genet.* 38 228–233 10.1038/ng1725 16380711 PMC2538576

[B11] Chen J. Y. Hsu H. C. Lee C. S. Chen D. S. Zuckerman A. J. Harrison T. J. (1988). Detection of hepatitis B virus DNA in hepatocellular carcinoma: methylation of integrated viral DNA. *J. Virol. Methods* 19 257–263 10.1016/0166-0934(88)90020-1 2836461

[B12] Chen Y. Song Y. X. Wang Z. N. (2013). The microRNA-148/152 family: multi-faceted players. *Mol. Cancer* 12 43 10.1186/1476-4598-12-43 PMC3671164 23683438

[B13] Chuang J. C. Jones P. A. (2007). Epigenetics and microRNAs. *Pediatr. Res.* 61 24R–29R 10.1203/pdr.0b013e3180457684 17413852

[B14] Cougot D. Allemand E. Riviere L. Benhenda S. Duroure K. Levillayer F. (2012). Inhibition of PP1 phosphatase activity by HBx: a mechanism for the activation of hepatitis B virus transcription. *Sci. Signal.* 5 ra1 10.1126/scisignal.2001906 22215732

[B15] Dakhlallah D. Batte K. Wang Y. Cantemir-Stone C. Z. Yan P. Nuovo G. (2013). Epigenetic regulation of miR-17~92 contributes to the pathogenesis of pulmonary fibrosis. *Am. J. Respir. Crit. Care Med.* 187 397–405 10.1164/rccm.201205-0888OC 23306545 PMC3603596

[B16] Datta J. Kutay H. Nasser M. W. Nuovo G. J. Wang B. Majumder S. (2008). Methylation mediated silencing of MicroRNA-1 gene and its role in hepatocellular carcinogenesis. *Cancer Res.* 68 5049–5058 10.1158/0008-5472.CAN-07-6655 18593903 PMC2562630

[B17] Dienstag J. L. (2008). Hepatitis B virus infection. *N. Engl. J. Med.* 359 1486–1500 10.1056/NEJMra0801644 18832247

[B18] Gao P. Wong C. C. Tung E. K. Lee J. M. Wong C. M. Ng I. O. (2011). Deregulation of microRNA expression occurs early and accumulates in early stages of HBV-associated multistep hepatocarcinogenesis. *J. Hepatol.* 54 1177–1184 10.1016/j.jhep.2010.09.023 21145831

[B19] Gao W. Hu J. (2007). Formation of hepatitis B virus covalently closed circular DNA: removal of genome-linked protein. *J. Virol.* 81 6164–6174 10.1128/JVI.02721-06 17409153 PMC1900077

[B20] Gong Q. Chen S. Guo J. Sun H. Zheng G. Liu Q. (2011). Chromosome remodeling related to hepatitis B virus replication in HepG2 cells. *DNA Cell Biol.* 30 347–354 10.1089/dna.2010.1172 21345131

[B21] Griffiths-Jones S. Saini H. K. Van Dongen S. Enright A. J. (2008). miRBase: tools for microRNA genomics. *Nucleic Acids Res.* 36 D154–D158 10.1093/nar/gkm952 17991681 PMC2238936

[B22] Guo H. Liu H. Mitchelson K. Rao H. Luo M. Xie L. (2011). MicroRNAs-372/373 promote the expression of hepatitis B virus through the targeting of nuclear factor I/B. *Hepatology* 54 808–819 10.1002/hep.24441 21608007

[B23] Guo Y. Li Y. Mu S. Zhang J. Yan Z. (2009). Evidence that methylation of hepatitis B virus covalently closed circular DNA in liver tissues of patients with chronic hepatitis B modulates HBV replication. *J. Med. Virol.* 81 1177–1183 10.1002/jmv.21525 19475606

[B24] Hayes C. N. Akamatsu S. Tsuge M. Miki D. Akiyama R. Abe H. (2012). Hepatitis B virus-specific miRNAs and Argonaute2 play a role in the viral life cycle. *PLoS ONE* 7:e47490 10.1371/journal.pone.0047490 PMC3472984 23091627

[B25] Hou J. Lin L. Zhou W. Wang Z. Ding G. Dong Q. (2011). Identification of miRNomes in human liver and hepatocellular carcinoma reveals miR-199a/b-3p as therapeutic target for hepatocellular carcinoma. *Cancer Cell* 19 232–243 10.1016/j.ccr.2011.01.001 21316602

[B26] Hu W. Wang X. Ding X. Li Y. Zhang X. Xie P. (2013). MicroRNA-141 represses HBV replication by targeting PPARA. *PLoS ONE* 7:e34165 10.1371/journal.pone.0034165 PMC3316618 22479552

[B27] Huang J. Wang Y. Guo Y. Sun S. (2010). Down-regulated microRNA-152 induces aberrant DNA methylation in hepatitis B virus-related hepatocellular carcinoma by targeting DNA methyltransferase 1. *Hepatology* 52 60–70 10.1002/hep.23660 20578129

[B28] Iorio M. V. Piovan C. Croce C. M. (2010). Interplay between microRNAs and the epigenetic machinery: an intricate network. *Biochim. Biophys. Acta* 1799 694–701 10.1016/j.bbagrm.2010.05.005 20493980

[B29] Jin J. Tang S. Xia L. Du R. Xie H. Song J. (2013). MicroRNA-501 promotes HBV replication by targeting HBXIP. *Biochem. Biophys. Res. Commun.* 430 1228–1233 10.1016/j.bbrc.2012.12.071 23266610

[B30] Jung Y. J. Kim J. W. Park S. J. Min B. Y. Jang E. S. Kim N. Y. (2013). c-Myc-mediated overexpression of miR-17-92 suppresses replication of hepatitis B virus in human hepatoma cells. *J. Med. Virol.* 85 969–978 10.1002/jmv.23534 23532756

[B31] Kim J. W. Lee S. H. Park Y. S. Hwang J. H. Jeong S. H. Kim N. (2011). Replicative activity of hepatitis B virus is negatively associated with methylation of covalently closed circular DNA in advanced hepatitis B virus infection. *Intervirology* 54 316–325 10.1159/000321450 21242658

[B32] Kong G. Zhang J. Zhang S. Shan C. Ye L. Zhang X. (2011). Upregulated microRNA-29a by hepatitis B virus X protein enhances hepatoma cell migration by targeting PTEN in cell culture model. *PLoS ONE* 6:e19518 10.1371/journal.pone.0019518 PMC3088678 21573166

[B33] Levrero M. Pollicino T. Petersen J. Belloni L. Raimondo G. Dandri M. (2009). Control of cccDNA function in hepatitis B virus infection. *J. Hepatol.* 51 581–592 10.1016/j.jhep.2009.05.022 19616338

[B34] Liu A. M. Zhang C. Burchard J. Fan S. T. Wong K. F. Dai H. (2011). Global regulation on microRNA in hepatitis B virus-associated hepatocellular carcinoma. *OMICS* 15 187–191 10.1089/omi.2010.0098 21319996

[B35] Lutgehetmann M. Volz T. Kopke A. Broja T. Tigges E. Lohse A.W. (2010). *In vivo* proliferation of hepadnavirus-infected hepatocytes induces loss of covalently closed circular DNA in mice. *Hepatology* 52 16–24 10.1002/hep.23611 20578126

[B36] Mailliard M. E. Gollan J. L. (2006). Emerging therapeutics for chronic hepatitis B. *Annu. Rev. Med.* 57 155–166 10.1146/annurev.med.57.121304.131422 16409142

[B37] McMahon B. J. (2009). The natural history of chronic hepatitis B virus infection. *Hepatology* 49 S45–S55 10.1007/s12072-008-9112-z 19399792

[B38] Miller R. H. Robinson W. S. (1983). Integrated hepatitis B virus DNA sequences specifying the major viral core polypeptide are methylated in PLC/PRF/5 cells. *Proc. Natl. Acad. Sci. U.S.A.* 80 2534–2538 10.1073/pnas.80.9.2534 6302693 PMC393860

[B39] Mogul D. Torbenson M. Schwarz K. B. (2011). Epigenetic regulation of hepatitis B virus infection. *Curr. Hepat. Rep.* 10 277–284 10.1007/s11901-011-0113-3

[B40] Murray J. M. Wieland S. F. Purcell R. H. Chisari F. V. (2005). Dynamics of hepatitis B virus clearance in chimpanzees. *Proc. Natl. Acad. Sci. U.S.A.* 102 17780–17785 10.1073/pnas.0508913102 16306261 PMC1345724

[B41] Newbold J. E. Xin H. Tencza M. Sherman G. Dean J. Bowden S. (1995). The covalently closed duplex form of the hepadnavirus genome exists *in situ* as a heterogeneous population of viral minichromosomes. *J. Virol.* 69 3350–3357 7745682 10.1128/jvi.69.6.3350-3357.1995 PMC189047

[B42] Noonan E. J. Place R. F. Pookot D. Basak S. Whitson J. M. Hirata H. (2009). miR-449a targets HDAC-1 and induces growth arrest in prostate cancer. *Oncogene* 28 1714–1724 10.1038/onc.2009.19 19252524

[B43] Novellino L. Rossi R. L. Bonino F. Cavallone D. Abrignani S. Pagani M. (2012). Circulating hepatitis B surface antigen particles carry hepatocellular microRNAs. *PLoS ONE* 7:e31952 10.1371/journal.pone.0031952 PMC3314627 22470417

[B44] Palumbo G. Belloni L. Valente S. Rotili D. Pediconi N. Mai A. (2013). Suppression of hepatitis B virus (HBV) transcription and replication by small molecules that target the epigenetic control of nuclear cccDNA minichromosome. *J. Hepatol.* 58 S25 10.1016/S0168-8278(13)60058-6

[B45] Pardo M. Bartolome J. Carreno V. (2007). Current therapy of chronic hepatitis B. *Arch. Med. Res.* 38 661–677 10.1016/j.arcmed.2006.12.013 17613358

[B46] Pollicino T. Belloni L. Raffa G. Pediconi N. Squadrito G. Raimondo G. (2006). Hepatitis B virus replication is regulated by the acetylation status of hepatitis B virus cccDNA-bound H3 and H4 histones. *Gastroenterology* 130 823–837 10.1053/j.gastro.2006.01.001 16530522

[B47] Potenza N. Papa U. Mosca N. Zerbini F. Nobile V. Russo A. (2011). Human microRNA hsa-miR-125a-5p interferes with expression of hepatitis B virus surface antigen. *Nucleic Acids Res.* 39 5157–5163 10.1093/nar/gkr067 21317190 PMC3130258

[B48] Quasdorff M. Protzer U. (2010). Control of hepatitis B virus at the level of transcription. *J. Viral Hepat.* 17 527–536 10.1111/j.1365-2893.2010.01315.x 20546497

[B49] Rivenbark A. G. Stolzenburg S. Beltran A. S. Yuan X. Rots M. G. Strahl B. D. (2012). Epigenetic reprogramming of cancer cells via targeted DNA methylation. *Epigenetics* 7 350–360 10.4161/epi.19507 22419067 PMC3368819

[B50] Schulze A. Gripon P. Urban S. (2007). Hepatitis B virus infection initiates with a large surface protein-dependent binding to heparan sulfate proteoglycans. *Hepatology* 46 1759–1768 10.1002/hep.21896 18046710

[B51] Scisciani C. Belloni L. Guerrieri F. Levrero M. Pediconi N. (2011). mir-224 is a direct target of hbx and modulates hbv replication. *J. Hepatol.* 54 S444 10.1016/S0168-8278(11)61123-9

[B52] Seeger C. Mason W. S. (2000). Hepatitis B virus biology. *Microbiol. Mol. Biol. Rev.* 64 51–68 10.1128/MMBR.64.1.51-68.2000 10704474 PMC98986

[B53] Skalsky R. L. Cullen B. R. (2010). Viruses, microRNAs, and host interactions. *Annu. Rev. Microbiol.* 64 123–141 10.1146/annurev.micro.112408.134243 20477536 PMC3621958

[B54] Song K. Han C. Zhang J. Lu D. Dash S. Feitelson M. (2013). Epigenetic regulation of miR-122 by PPARgamma and hepatitis B virus X protein in hepatocellular carcinoma cells. *Hepatology*. 10.1002/hep.26514 [Epub ahead of print] PMC3773012 23703729

[B55] Su C. Hou Z. Zhang C. Tian Z. Zhang J. (2011). Ectopic expression of microRNA-155 enhances innate antiviral immunity against HBV infection in human hepatoma cells. *Virol. J.* 8 354 10.1186/1743-422X-8-354 PMC3169510 21762537

[B56] Tian Y. Yang W. Song J. Wu Y. Ni B. (2013). Hepatitis B virus x protein-induced aberrant epigenetic modifications contributing to human hepatocellular carcinoma pathogenesis. *Mol. Cell. Biol.* 33 2810–2816 10.1128/MCB.00205-13 23716588 PMC3719687

[B57] Ura S. Honda M. Yamashita T. Ueda T. Takatori H. Nishino R. (2009). Differential microRNA expression between hepatitis B and hepatitis C leading disease progression to hepatocellular carcinoma. *Hepatology* 49 1098–1112 10.1002/hep.22749 19173277

[B58] Vivekanandan P. Daniel H. D. Kannangai R. Martinez-Murillo F. Torbenson M. (2010). Hepatitis B virus replication induces methylation of both host and viral DNA. *J. Virol.* 84 4321–4329 10.1128/JVI.02280-09 20147412 PMC2863779

[B59] Vivekanandan P. Kannangai R. Ray S. C. Thomas D. L. Torbenson M. (2008a). Comprehensive genetic and epigenetic analysis of occult hepatitis B from liver tissue samples. *Clin. Infect. Dis.* 46 1227–1236 10.1086/529437 18444860 PMC3140175

[B60] Vivekanandan P. Thomas D. Torbenson M. (2008b). Hepatitis B viral DNA is methylated in liver tissues. *J. Viral Hepat.* 15 103–107 10.1111/j.1365-2893.2007.00905.x 18184192

[B61] Vivekanandan P. Thomas D. Torbenson M. (2009). Methylation regulates hepatitis B viral protein expression. *J. Infect. Dis.* 199 1286–1291 10.1086/597614 19301974 PMC3032270

[B62] Wang S. Qiu L. Yan X. Jin W. Wang Y. Chen L. (2012a). Loss of microRNA 122 expression in patients with hepatitis B enhances hepatitis B virus replication through cyclin G(1)-modulated P53 activity. *Hepatology* 55 730–741 10.1002/hep.24809 22105316

[B63] Wang W. Zhao L. J. Tan Y. X. Ren H. Qi Z. T. (2012b). Identification of deregulated miRNAs and their targets in hepatitis B virus-associated hepatocellular carcinoma. *World J. Gastroenterol.* 18 5442–5453 10.3748/wjg.v18.i38.5442 23082062 PMC3471114

[B64] Wang Y. Jiang L. Ji X. Yang B. Zhang Y. Fu X. D. (2013a). Hepatitis B viral RNA directly mediates down-regulation of the tumor suppressor microRNA miR-15a/miR-16-1 in hepatocytes. *J. Biol. Chem.* 288 18484–18493 10.1074/jbc.M113.458158 23649629 PMC3689990

[B65] Wang Y. Toh H. C. Chow P. Chung A. Y. Meyers D. J. Cole P. A. (2013b). MicroRNA-224 is up-regulated in hepatocellular carcinoma through epigenetic mechanisms. *FASEB J.* 26 3032–3041 10.1096/fj.11-201855 22459148 PMC3382089

[B66] Wang Y. Lu Y. Toh S. T. Sung W. K. Tan P. Chow P. (2010). Lethal-7 is down-regulated by the hepatitis B virus x protein and targets signal transducer and activator of transcription 3. *J. Hepatol.* 53 57–66 10.1016/j.jhep.2009.12.043 20447714

[B67] Wei X. Tan C. Tang C. Ren G. Xiang T. Qiu Z. (2013a). Epigenetic repression of miR-132 expression by the hepatitis B virus x protein in hepatitis B virus-related hepatocellular carcinoma. *Cell. Signal.* 25 1037–1043 10.1016/j.cellsig.2013.01.019 23376496

[B68] Wei X. Xiang T. Ren G. Tan C. Liu R. Xu X. (2013b). miR-101 is down-regulated by the hepatitis B virus x protein and induces aberrant DNA methylation by targeting DNA methyltransferase 3A. *Cell. Signal.* 25 439–446 10.1016/j.cellsig.2012.10.013 23124077

[B69] Werle-Lapostolle B. Bowden S. Locarnini S. Wursthorn K. Petersen J. Lau G. (2004). Persistence of cccDNA during the natural history of chronic hepatitis B and decline during adefovir dipivoxil therapy. *Gastroenterology* 126 1750–1758 10.1053/j.gastro.2004.03.018 15188170

[B70] Wu G. Yu F. Xiao Z. Xu K. Xu J. Tang W. (2011). Hepatitis B virus X protein downregulates expression of the miR-16 family in malignant hepatocytes *in vitro*. *Br. J. Cancer* 105 146–153 10.1038/bjc.2011.190 21629246 PMC3137408

[B71] Xu X. Fan Z. Kang L. Han J. Jiang C. Zheng X. (2013). Hepatitis B virus X protein represses miRNA-148a to enhance tumorigenesis. *J. Clin. Invest.* 123 630–645 10.1172/JCI64265 23321675 PMC3561812

[B72] Yan H. Zhong G. Xu G. He W. Jing Z. Gao Z. (2012). Sodium taurocholate cotransporting polypeptide is a functional receptor for human hepatitis B and D virus. *eLife* 1 e00049 10.7554/eLife.00049 PMC3485615 23150796

[B73] Zhang G. L. Li Y. X. Zheng S. Q. Liu M. Li X. Tang H. (2010). Suppression of hepatitis B virus replication by microRNA-199a-3p and microRNA-210. *Antiviral Res.* 88 169–175 10.1016/j.antiviral.2010.08.008 20728471

[B74] Zhang X. Chen X. Lin J. Lwin T. Wright G. Moscinski L. C. (2013a). Myc represses miR-15a/miR-16-1 expression through recruitment of HDAC3 in mantle cell and other non-Hodgkin B-cell lymphomas. *Oncogene* 31 3002–3008 10.1038/onc.2011.470 22002311 PMC3982396

[B75] Zhang Y. Li C. Zhu H. Kang Y. Liu H. Wang J. (2013b). Comparative analysis of CpG islands among HBV genotypes. *PLoS ONE* 8:e56711 10.1371/journal.pone.0056711 PMC3579858 23451072

[B76] Zhang X. Liu S. Hu T. He Y. Sun S. (2009). Up-regulated microRNA-143 transcribed by nuclear factor kappa B enhances hepatocarcinoma metastasis by repressing fibronectin expression. *Hepatology* 50 490–499 10.1002/hep.23008 19472311

[B77] Zhang X. Zhang E. Ma Z. Pei R. Jiang M. Schlaak J. F. (2011). Modulation of hepatitis B virus replication and hepatocyte differentiation by microRNA-1. *Hepatology* 53 1476–1485 10.1002/hep.24195 21520166

[B78] Zoulim F. (2005). New insight on hepatitis B virus persistence from the study of intrahepatic viral cccDNA. *J. Hepatol.* 42 302–308 10.1016/j.jhep.2004.12.015 15710212

